# The Two-Domain Model Utilizing the Effective Pinning Energy for Modeling the Strain-Dependent Magnetic Permeability in Anisotropic Grain-Oriented Electrical Steels

**DOI:** 10.3390/ma17020369

**Published:** 2024-01-11

**Authors:** Tadeusz Szumiata, Paweł Rekas, Małgorzata Gzik-Szumiata, Michał Nowicki, Roman Szewczyk

**Affiliations:** 1 Faculty of Mechanical Engineering, Department of Physics, Kazimierz Pulaski Radom University, Stasieckiego 54, 26-600 Radom, Poland; t.szumiata@uthrad.pl (T.S.); m.gzik@uthrad.pl (M.G.-S.); 2 Faculty of Mechatronics, Warsaw University of Technology, Sw. Andrzeja Boboli 8, 02-525 Warsaw, Poland; pawel.rekas.dokt@pw.edu.pl; 3 Department of Mechatronics, Robotics and Digital Manufacturing, Faculty of Mechanics, Vilnius Gediminas Technical University, Plytinės g. 25, LT-10105 Vilnius, Lithuania; michal.nowicki@vilniustech.lt

**Keywords:** magnetoelastic effect, magnetic anisotropy, electrical steels

## Abstract

This paper presents a newly proposed domain wall energy-based model of the 2D strain dependence of relative magnetic permeability in highly grain-oriented anisotropic electrical steels. The model was verified utilizing grain-oriented M120-27s electrical steel sheet samples with magnetic characteristics measured by an automated experimental setup with a magnetic yoke. The model’s parameters, identified in the differential evolution-based optimization process, enable a better understanding of the interaction between stress-induced anisotropy and magnetocrystalline anisotropy in electrical steels. Moreover, the consequences of the simplified description of grain-oriented magnetocrystalline anisotropy are clearly visible, which opens up the possibility for further research to improve this description.

## 1. Introduction

The influence of mechanical strain caused by the stresses from external forces on the magnetic characteristics of soft magnetic materials is very important from both theoretical [1] and applied [2,3] points of view. In spite of the fact that these different physical 3D models of the influence of strain tensor on the relative magnetic permeability *μ_r_* tensor were presented previously [4,5], the generalized model of the influence of mechanical stresses on the magnetic characteristics of anisotropic materials was still not presented.

It should be highlighted that recently developed advanced experimental setups for testing the 2D mechanical stress dependence of relative magnetic permeability in electrical steels [6,7,8] have brought a fresh impetus into the process of understanding the magnetoelastic phenomena in strongly anisotropic soft magnetic materials. On the other hand, it should be highlighted that the interaction between magnetocrystalline anisotropy and stress-induced anisotropy requires an explanation on the basis of quantum physics phenomena. Simplified models utilizing a physical explanation of axial stress-induced anisotropy [9,10,11] or grain-oriented anisotropy [12] are not valid in the case of the interaction between magnetoelastic and magnetocrystalline anisotropy.

It should also be highlighted that models of the stress dependence of functional characteristics of magnetic materials are essential from the point of view of power conversion devices, especially power transformers. Such models create the possibility of understanding the operation of real devices in harsh operating conditions where mechanical stresses can be accidentally applied to the transformer core during the device installation, or due to thermal expansion. As a result, mechanical stresses change the characteristics of magnetic materials, such as electrical steels, which may reduce power conversion efficiency or even cause the whole system to malfunction. It should also be highlighted that the model of stress dependence of 2D relative permeability of the transformer’s core material enables the development of finite elements-based models describing the operation of power conversion devices under the influence of mechanical stresses.

On the other hand, the main scientific motivation for this presented work was to find out if the macroscopically measured anisotropy of magnetic permeability in grain-oriented steel can be explained in terms of the microscopic anisotropy of domain wall pinning energy. For this purpose, the model should be precise enough to reproduce anisotropy, but on the other hand, sufficiently simple and numerically effective to enable the fitting to the experimental data, which requires multiple recalculations. The origin of domain wall pinning energy seems to be poorly recognized. Due to the planar geometry of thin films or rolled ribbons (sheets), one can expect Bloch-type domain walls with an in-plain spin direction. Due to the continuous distribution of spin direction in the domain wall, at first glance, one should not expect any anisotropy of pinning energy even in rolled and strained steel elements. However, the key factor responsible for such anisotropy could be the magnetoelastic coupling of individual spins with a crystalline lattice, which is the case for the materials with a non-zero magnetostriction constant. When the material is strained or rolled, the wells of pinning potential are deformed, and at the same time, the spin helicoid in the domain wall undergoes deformation due to the magnetoelastic deformation. This leads to the anisotropy of pinning potential, and that is why magnetizing efficiency depends on the in-plane direction of the applied magnetic field relative to the strain axis. Since our model and experiment referred to the low-field regime, not rotations of the spin directions in coupled domains related to magnetocrystalline anisotropy, only domain wall motion was considered the dominating mechanism of magnetization. This effect, in general, is strain-dependent.

This paper tries to approach filling this gap in the state of the field pertaining to the lack of the model connecting the strain dependence of anisotropy of magnetic materials. The proposed domain wall energy-based model explains the 2D strain dependence of relative magnetic permeability in highly grain-oriented anisotropic electrical steels. Moreover, the parameters of the model identified in the differential evolution-based optimization process enable a better understanding of the consequences of the simplified description of grain-oriented anisotropy in electrical steels. 

The stress dependence of the magnetic permeability can be described theoretically with the extension of the model presented in [13]. In this previous “one domain” approach, the domain wall motion was considered exclusively via an effective, modified magneto-crystalline anisotropy energy constant. However, more precise analysis in the case of grain-oriented electrical steels requires explicit incorporation of domain wall pinning phenomena [14,15,16]. Currently, a deeper understanding and control of domain wall pinning processes is a subject of research that is often reported in *Nature* journals, e.g., [17], due to possible applications in the construction of magnetic memories. The most advanced and recent method of domain wall motion description is a theoretical concept of pinning field distributions [18]. Nevertheless, in our work, the main focus was on the impact of the pinning potential anisotropy on the magnetic permeability.

## 2. Proposed Model

**Figure 1:**
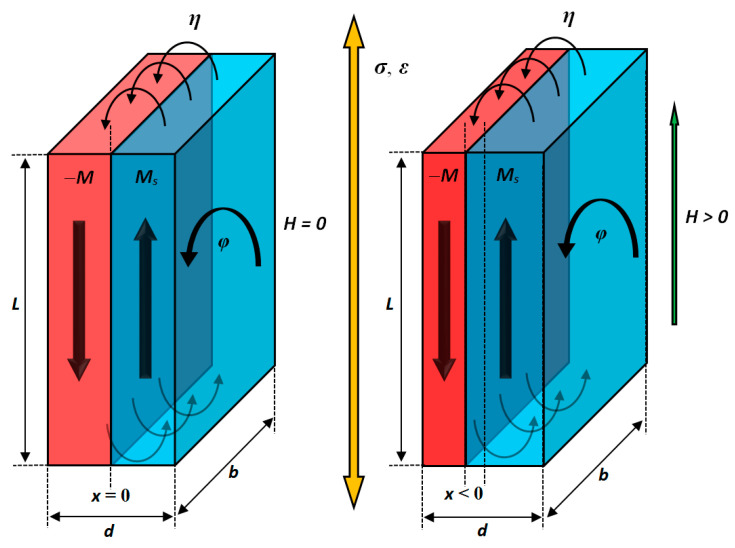
Magnetization process scheme of two-domain system with anisotropic, stress-dependent pinning energy of domain wall.

To predict the low-field magnetic permeability of grain-oriented electrical steels, the two-domain system with anisotropic, stress-dependent pinning energy of the domain wall was considered, as presented in Figure 1. The in-plane saturation magnetization $M_{s}$ in the adjacent domains has the opposite directions to reduce the energy of the mutual magnetostatic interaction. After applying external magnetic field H in the plane, the volume of the domain with a magnetization direction in line with the field increases. In the example seen in Figure 1, this corresponds to wall domain movement to the left (*x* < 0). The value of *x* shift depends on the external magnetic field’s strength and the domain wall’s pinning energy. Due to the rolling of grain-oriented electrical steels, the pinning energy reveals the anisotropy (i.e., the dependence on the in-plane *φ* angle between the rolling direction and the direction of the external magnetic field followed by magnetization). The anisotropy of pinning energy augments when external stress *σ* is applied, causing the strain *ε* of the material and changing the interatomic distances.

The density of dipolar magnetic energy of the domain interaction with the external magnetic field of the strength *H* is equal to:

(1) $$E_{dip}=-M_{s}B_{0}\cos\left(\theta \right)$$

where $B_{0}=\mu_{0}H$ is the value of the induction of the external magnetic field and θ denotes the angle between the saturation magnetization and external magnetic field. 

In the case of the considered system of two domains (with magnetization parallel and antiparallel to the external magnetic field), the average density of magnetic energy can be expressed as:

(2) $$E_{B_{0}}\left(x\right)=\frac{-M_{s}B_{0}L\left(\frac{d}{2}-x\right)b+M_{s}B_{0}L\left(\frac{d}{2}+x\right)b}{Ldb}$$

where Ldb is the volume of the system (L, d and b dimensions marked in Figure 1). This takes a simplified form:

(3) $$E_{B_{0}}\left(x\right)=2B_{0}M_{s}\frac{x}{d}$$

In order to estimate the average energy density $E_{ms}\left(x\right)$ of the two domains’ magnetostatic interaction, it was assumed that the magnetic moment of one domain is the source of the magnetic field of antiparallel direction to the magnetization in the outer region. The magnetic moment of the second domain is immersed in this field; thus, $E_{ms}\left(x\right)$ can be written as:

(4) $$E_{ms}\left(x\right)=-\eta \mu_{0}M_{s}\frac{L\left(\frac{d}{2}-x\right)b}{Ldb}M_{s}\frac{L\left(\frac{d}{2}+x\right)b}{Ldb}\;$$

where $\mu_{0}$ is the magnetic permeability of the vacuum and η is the dimensionless coupling parameter that is—in general—dependent on the size and shape of the system. Formula (4) simplifies to:

(5) $$E_{ms}\left(x\right)=-\eta \mu_{0}M_{s}^{2}\left[\frac{1}{4}-{\left(\frac{x}{d}\right)}^{2}\right]$$

Stress-dependent, anisotropic density of pinning energy has been modelled as a simple quadratic function of domain wall displacement x from equilibrium point, x=0 (which corresponds at the same time to the minimum of $E_{B_{0}}$ and $E_{ms}$ energies for H=0):

(6) $$E_{pin}\left(x,\varphi ,\sigma \right)=\frac{1}{2}P\left(\varphi ,\sigma \right){\left(\frac{x}{d}\right)}^{2}$$

where $P\left(\varphi ,\sigma \right)$ is the phenomenological parameter expressed in the units of energy density and—in general—depending on stress and the magnetization direction.

The density of the total energy of the system is a sum of the three mentioned constituents:

(7) $$E\left(x,\varphi ,\sigma \right)=E_{B_{0}}\left(x\right)+E_{ms}\left(x\right)+E_{pin}\left(x,\varphi ,\sigma \right)$$

In the equilibrium state (thermally nonperturbed), the energy takes a minimum value. Calculating its first derivative with respect to x:

(8) $$\frac{\partial }{\partial x}E\left(x,\varphi ,\sigma \right)=0$$

and considering definitions (3), (5) and (6), one obtains the following formula for the position of the domain wall:

(9) $$\frac{x}{d}=\frac{-2B_{0}M_{s}}{P\left(\varphi ,\sigma \right)+2\eta \mu_{0}M_{s}^{2}}$$

Balancing the total magnetic moment of both domains, it is possible to write an average magnetization of the system in the following way:

(10) $$\left(x,\varphi ,\sigma \right)=\frac{M_{s}\left(\frac{d}{2}-x\right)-M_{s}\left(\frac{d}{2}+x\right)}{d}=-2M_{s}\frac{x}{d}$$

Inserting Formula (9) into (10) produces the dependence of the average magnetization on the applied field of induction $B_{0}$:

(11) $$M\left(B_{0},\varphi ,\sigma \right)=\frac{4B_{0}M_{s}^{2}}{P\left(\varphi ,\sigma \right)+2\eta \mu_{0}M_{s}^{2}}$$

When using the strength of external magnetic field H, the formula can be rewritten as:

(12) $$M\left(H,\sigma ,\varphi \right)=\frac{4H\mu_{0}M_{s}^{2}}{P\left(\varphi ,\sigma \right)+2\eta \mu_{0}M_{s}^{2}}$$

Low-field DC magnetic susceptibility is defined as follows:

(13) $$\chi \left(\varphi ,\sigma \right)\equiv {\left.\frac{\partial }{\partial H}M\left(H,\varphi ,\sigma \right)\right|}_{H=0}$$

Considering Equation (12), one obtains the final form:

(14) $$\chi \left(\varphi ,\sigma \right)=\frac{4\mu_{0}M_{s}^{2}}{P\left(\varphi ,\sigma \right)+2\eta \mu_{0}M_{s}^{2}}$$

Thus, the relative magnetic permeability of the material is equal to:

(15) $$\mu_{r}\left(\varphi ,\sigma \right)=1+\chi \left(\varphi ,\sigma \right)=1+\frac{1}{\frac{P\left(\varphi ,\sigma \right)}{4\mu_{0}M_{s}^{2}}+\frac{1}{2}\eta }$$

To reproduce a strain-dependent anisotropy of sufficiently high order, it was assumed that the phenomenological parameter $P\left(\varphi ,\sigma \right)$ of the parabolic well of domain wall pinning energy takes the following form:

(16) $$P\left(\varphi ,\sigma \right)\equiv P_{∥}\left(\sigma \right){\cdot cos}^{2}\varphi +P_{⊥}\left(\sigma \right){\cdot sin}^{2}\varphi +Q_{⊥}\left(\sigma \right)\cdot {sin}^{6}\varphi$$

where $P_{∥}\left(\sigma \right),\;{\;P}_{⊥}\left(\sigma \right)$ and $Q_{⊥}\left(\sigma \right)$ are the parameters of the model. This leads to the following anisotropy of the strain-dependent relative magnetic permeability:

(17) $$\mu_{r}\left(\varphi ,\sigma \right)=1+\frac{1}{\frac{P_{∥}\left(\sigma \right)}{4\mu_{0}M_{s}^{2}}{\cdot cos}^{2}\varphi +\frac{P_{⊥}\left(\sigma \right)}{4\mu_{0}M_{s}^{2}}{\cdot sin}^{2}\varphi +\frac{Q_{⊥}\left(\sigma \right)}{4\mu_{0}M_{s}^{2}}\cdot {sin}^{6}\varphi +\frac{1}{2}\eta }$$

Introducing normalized, dimensionless parameters $p_{∥}\left(\sigma \right),\;{\;p}_{⊥}\left(\sigma \right)$ and $q_{⊥}\left(\sigma \right)$ of pinning energy of domain walls, one obtains the final formula for the anisotropic, stress-dependent relative magnetic permeability of grain-oriented electrical steel:

(18) $$\mu_{r}\left(\varphi ,\sigma \right)=1+\frac{1}{p_{∥}\left(\sigma \right){\cdot cos}^{2}\varphi +p_{⊥}\left(\sigma \right){\cdot sin}^{2}\varphi +q_{⊥}\left(\sigma \right)\cdot {sin}^{6}\varphi +\frac{1}{2}\eta }\;$$

In the real multidomain system at low magnetic fields (far from the saturation state), the coupling constant of magnetostatic interaction can be omitted (η→0)—especially for the case of the elongated shape of the domains (L≫d). Under such an assumption, Formula (18) was applied to fit the experimental data.

The form of the $P\left(\varphi ,\sigma \right)$ parameter was chosen phenomenologically after preliminary analysis of the experimental data concerning measured $\mu_{r}\left(\varphi ,\sigma \right)$ dependencies. There is no simple analogy between the magnetocrystalline anisotropy and the anisotropy of domain walls’ pinning energy. There is an entirely different mechanism of the stress impact. The magnetocrystalline anisotropy varies due to magnetostrictive effects, whereas pinning energy changes due to the movement of crystalline lattice defects along the axis of rolling and the direction of the external stress. This could be an explanation of the fact that the uniaxial anisotropy of the pinning energy dominates, and that there is no fourth-order term typical for magnetocrystalline anisotropy in iron or ferritic non-rolled steels. The sixth-order term (with non-zero $Q_{⊥}\left(\sigma \right)$ coefficient) is presumably responsible for the effect of the crystalline lattice defects’ shift in direction perpendicular to the stress. Nevertheless, this effect is less significant than the longitudinal movement of defects; thus, the expected values of $Q_{⊥}\left(\sigma \right)$ should be smaller than other coefficients in the case of small stress (which has been confirmed by experimental data analysis presented in Figure 3c).

## 3. Method of Experimental Investigation

**Figure 2:**
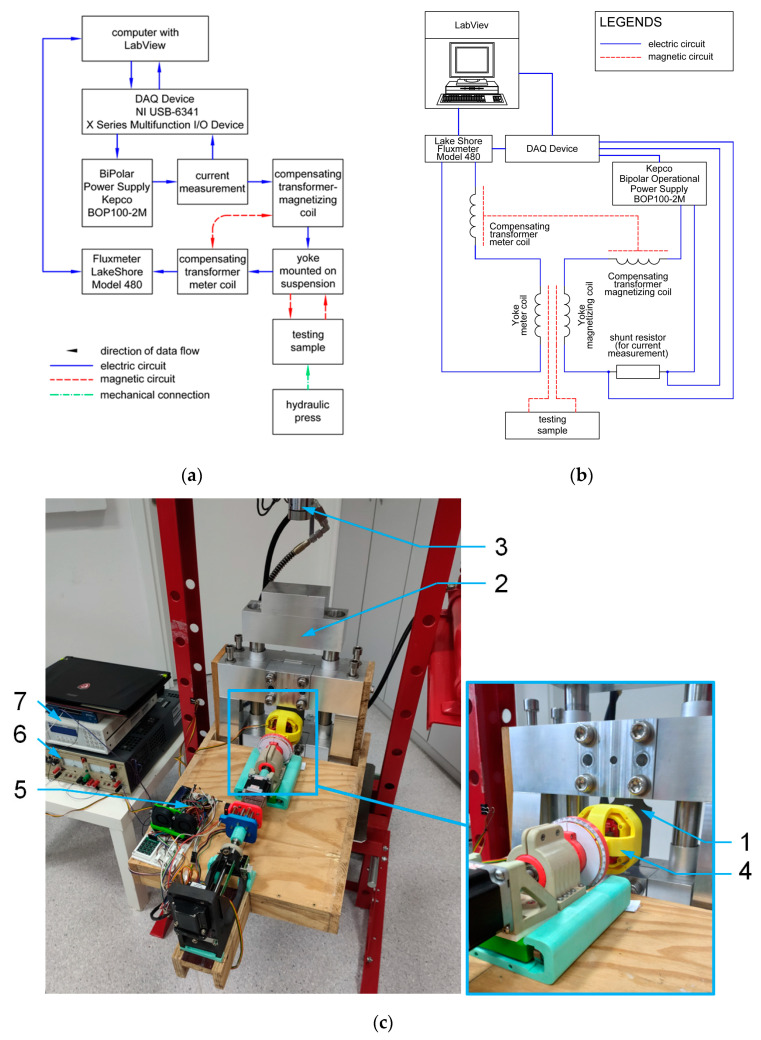
The experimental setup: (**a**) schematic block diagram, (**b**) schematic wiring system, (**c**) photography of experimental setup in operation: 1—tested sheet sample, 2—non-magnetic mechanical system, 3—hydraulic press, 4—yoke head with Cardan gimbal mechanism, 5—rotation mechanism, 6—yoke power supply, 7—fluxmeter.

The method of experimental investigation on the tensile stress *σ* dependence of 2D relative magnetic permeability *μ_r_* of grain-oriented M120-27s electrical steel was presented in detail in the previous paper [8]. The schematic block diagram of the experimental setup is presented in Figure 2a, its electrical connections are presented in Figure 2b, while its general view is presented in Figure 2c.

The measuring system was developed previously [8] at Warsaw University of Technology, Faculty of Mechatronics, in cooperation with the Industrial Research Institute for Automation and Measurements (Łukasiewicz Research Network). During the investigation, strip-shaped grain-oriented M120-27s electrical steel sheet samples (1) were used. The investigated sample consisted of three sections: two mounting sections and a measuring area. The shape of the samples was optimized on the basis of the finite elements modeling method to guarantee the uniform distribution of tensile stresses in the measuring area. This uniformity was guaranteed by special trimming of the strip-shaped sample together with the openings between the mounting and measuring sections [8].

Samples were mounted in the non-magnetic mechanical system (2), transforming the compressive forces from the hydraulic press (3) into tensile stresses in the sample. Due to the application of a force-reversing system, the proposed mechanical setup provided the possibility of testing both the tensile and compressive stress dependence of mechanical characteristics of the sample. However, the range of stresses during the compressive stress tests was strongly limited due to the risk of buckling in the case of the thin strip-shaped sample made of electrical steel.

The key problem connected with yoke-based measurements of magnetic properties of strip-shaped samples is the quality of contact between the yoke and the surface of the tested sample. For this reason, the specialized yoke head with a 2D Cardan gimbal mechanism (4), which guaranteed proper contact with the sample (1), was proposed [8]. As a result, the precise rotation of the yoke performed by a digitally controlled step motor mechanism (5) did not lead to the degradation of the metrological characteristic of the magnetic measurement system, and 2D relative permeability tests of the magnetic sample under stresses were possible. The yoke was made from high-saturation (1.9T) electrical steel sheets, allowing for an application of up to 2000 A/m H exciting fields.

The rotating yoke head with a 2D Cardan gimbal mechanism was connected with a digitally controlled hysteresis graph system based on a PC computer equipped with the DAQ NI-USB 6341 data acquisition and control card. The yoke was driven by the Kepco BOP-100-2M power supply (6), which operates as a highly efficient voltage-to-current converter. The relative magnetic permeability of the sample was measured by a Lakeshore 480 fluxmeter (7) with digitally corrected characteristics. The results of the measurements were visualized and archived in text files to simplify further processing.

The measurements were carried out at room temperature. The changes of temperature during the full cycle of measurements did not exceed 2 °C. As a result, the influence of temperature on the results of the measurements can be neglected.

It was estimated previously [8] that in the typical range of operation, the relative uncertainty of measurements in the presented system does not exceed 1%. However, special care should be taken to provide a good, high-quality sample surface and reduce the magnetic flux leakage.

## 4. Identification of the Model’s Parameters

The identification of parameters $p_{∥}$, $p_{⊥}$ and $q_{∥}$ in the model proposed in Equation (18) was performed in the optimization process. The target function of optimization *G* was defined as:

(19) $$G=\sum_{i=1}^{n}{\left(\mu_{r\;meas}\left(\phi_{i}\right)-\mu_{r\;model}\left(\phi_{i}\right)\;\right)}^{2}$$

where *μ_r meas_* and *μ_r model_* are the results of measurements and the results of modeling, respectively, for a given value of angle *ϕ_i_*. 

Due to the fact that it is expected that the target function exhibits local minima, the differential optimization algorithm [19] was utilized. Moreover, considering the fact that stresses *σ* were applied in the rolling axis direction and limited to the elastic range of deformation, the assumption of the linear stress dependence of parameters $p_{∥}\left(\sigma \right),\;{\;p}_{⊥}\left(\sigma \right)$ and $q_{⊥}\left(\sigma \right)$ was made. The model’s parameters identified during the optimization process are presented in Table 1.

**Figure 3:**
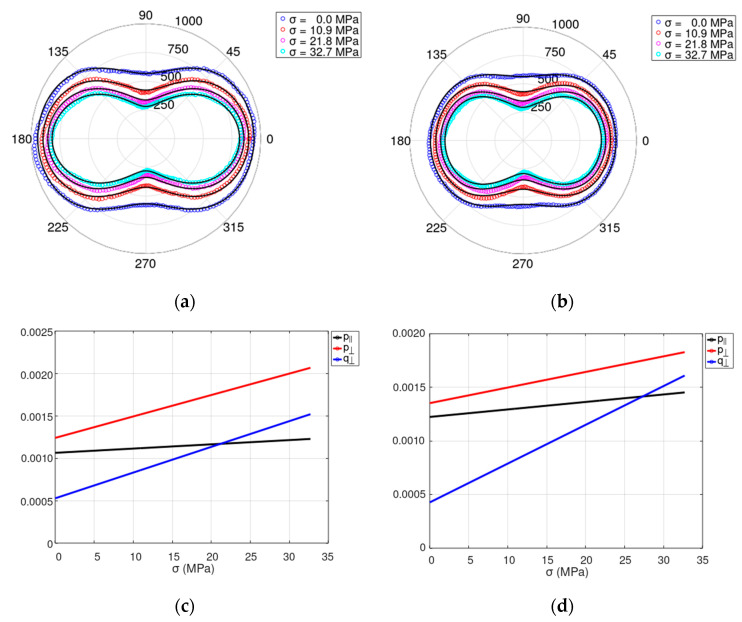
Results of measurements and modeling of the influence of mechanical stresses *σ* on the 2D relative permeability *μ_r_* for the sheet sample made of grain-oriented M120-27s electrical steel. Tensile stresses were applied on the 0°–180° axis: (**a**,**b**) The comparison of the experimental results (points) and the results of modeling (solid line) of the 2D plots of relative permeability *μ_r_* (dimensionless), for the magnetizing field amplitude of: (**a**) *H_m_* = 760 A/m, (**b**) *H_m_* = 1010 A/m. (**c**,**d**) Linear stress dependence of parameters $p_{∥}$, $p_{⊥}$ and $q_{⊥}$ (dimensionless parameters) for the magnetizing field amplitude of: (**c**) *H_m_* = 760 A/m, (**d**) *H_m_* = 1010 A/m.

Figure 3a,b present the results of modeling the mechanical stress *σ* dependence of relative permeability *μ_r_* measured (points) and model the amplitude of the magnetizing field *H_m_* equal to 750 A/m and 1010 A/m, respectively (solid line). Moreover, Figure 3c,d present the linear stress dependence of parameters $p_{∥}\left(\sigma \right),\;{\;p}_{⊥}\left(\sigma \right)$ and $q_{⊥}\left(\sigma \right)$ determined for the indicated amplitudes of the magnetizing field *H_m_*. 

The results of modeling confirm the assumption of the linear stress dependence of parameters $p_{∥}\left(\sigma \right),\;{\;p}_{⊥}\left(\sigma \right)$ and $q_{⊥}\left(\sigma \right)$ for tensile stresses applied in the electrical steel rolling direction and limited to the elastic range of deformation. The proposed model successfully reproduces the character of changes to the 2D relative magnetic permeability in grain-oriented M120-27s electrical steel subjected to mechanical tensile stresses. The adequate agreement between the experimental measurements and the results of modeling is confirmed by the R-squared coefficient, which exceeds 0.99 for all values of tensile stresses *σ*.

## 5. Conclusions

This paper explored the pinning energy of a domain wall-based model of mechanical stress dependence with relative magnetic permeability in anisotropic electrical steels. In the proposed normalized model, dimensionless parameters $p_{∥}\left(\sigma \right),\;{\;p}_{⊥}\left(\sigma \right)$ and $q_{⊥}\left(\sigma \right)$ of pinning energy of domain walls were considered. In addition, considering the fact that stresses *σ* were applied in the rolling axis direction and limited to the elastic range of deformation, the assumption of the linear stress dependence of parameters $p_{∥}\left(\sigma \right),\;{\;p}_{⊥}\left(\sigma \right)$ and $q_{⊥}\left(\sigma \right)$ was made.

To validate the proposed model, experimental measurements were carried out on the sample made of M120-27s electrical steel subjected to tensile stresses applied in the rolling direction. The measurements were performed on the computer-controlled automatic measuring setup, where the 2D Cardan gimbal mechanism guaranteed proper contact between the rotating head of the yoke and the tested sample.

The results of modeling, carried out on the basis of the experimental measurements, confirmed very satisfactory agreement between the proposed model and the experimental results. It was confirmed that the assumption of linear stress dependence of parameters $p_{∥}\left(\sigma \right),\;{\;p}_{⊥}\left(\sigma \right)$ and $q_{⊥}\left(\sigma \right)$ enables efficient reproduction of experimental tensile stress dependence of 2D relative permeability; this was confirmed by the R-squared coefficient, which exceeded 0.99 for all values of tensile stresses *σ*.

It should be highlighted that due to the very satisfactory agreement with the experimental results from 2D measurements of relative magnetic permeability of grain-oriented electrical steels subjected to tensile stresses, the proposed model might be utilized in the finite elements-based modeling of the operation of power transformers subjected to mechanical stresses. Such models are very important from the point of view of power application because they enable the possibility of understanding the operation of real devices in harsh operating conditions. During such operations, mechanical stresses can be accidentally applied to the transformer core, changing its functional characteristics and potentially leading to malfunction or a reduction in operation efficiency. 

On the other hand, the efficient application of the presented model also requires considering the influence of temperature on 2D measurements of relative magnetic permeability of grain-oriented electrical steels. As a result, developing the proposed model to cover both mechanical stress and temperature dependence of magnetic characteristics in electrical steels is the most promising direction for further work.

## Figures and Tables

**Table 1:** Parameters of the linear stress dependence model $\sigma \left(MPa\right)+b$ where parameters $p_{∥}\left(\sigma \right),\;{\;p}_{⊥}\left(\sigma \right)$ and $q_{⊥}\left(\sigma \right)$ were identified during the optimization process for the experimental measurements of the sample made of grain-oriented M120-27s electrical steel.

*Parameter*	*H_m_* = 760 *A*/*m*		*H_m_ =* 1010 *A*/*m*	
	*a*(1/*MPa*)	*b*	*a*(1/*MPa*)	*b*
$p_{∥}$	(5.006 ± 0.008) × 10^−6^	(1.0638 ± 0.0002) × 10^−3^	(6.99 ± 0.01) × 10^−6^	(1.2238 ± 0.0002) × 10^−3^
$p_{⊥}$	(2.527 ± 0.004) × 10^−5^	(1.2398 ± 0.0006) × 10^−3^	(1.453 ± 0.005) × 10^−5^	(1.3520 ± 0.0008) × 10^−3^
$q_{⊥}$	(3.036 ± 0.004) × 10^−5^	(5.273 ± 0.009) × 10^−4^	(3.618 ± 0.006) × 10^−5^	(4.275 ± 0.001) × 10^−4^

## Data Availability

Data are contained within the article.
